# Personal and Emotional Factors of Nursing Professionals Related to Coping with End-of-Life Care: A Cross-Sectional Study

**DOI:** 10.3390/ijerph18189515

**Published:** 2021-09-09

**Authors:** María Povedano-Jiménez, Carmen Ropero-Padilla, Miguel Rodriguez-Arrastia, María Paz García-Caro

**Affiliations:** 1 Nursing Department, Faculty of Health Sciences, University of Granada, Av. de la Ilustracion, 18071 Granada, Spain; mpazgc@ugr.es; 2 Granada-Metropolitan Healthcare District, Joaquin Eguaras, 18013 Granada, Spain; 3 Pre-Department of Nursing, Faculty of Health Sciences, Jaume I University, Av. Sos Baynat, 12071 Castellon de la Plana, Spain; ropero@uji.es (C.R.-P.); arrastia@uji.es (M.R.-A.); 4 Research Group CYS, Faculty of Health Sciences, Jaume I University, Av. Sos Baynat, 12071 Castellon de la Plana, Spain

**Keywords:** anxiety, clinical competence, cross-sectional study, end-of-life care, nursing, psychological resilience

## Abstract

The death of a patient can be a traumatic event, causing emotional and psychological distress in professional nurses and potentially hampering the quality of their care. Optimal self-perceived coping with death involves valuing these difficult situations as challenges and actively coping with work-related stress during the care of the dying patient. Thus, the aim of this study was to assess Spanish nurses’ self-perceived competence with patient death and investigate its relationship with their personality traits, anxiety and fear of death. A cross-sectional study based on a web-based survey was conducted. A sample of 534 Spanish nurses provided socio-demographic information and answered validated questionnaires. Most participants perceived their coping with death as optimal. Men and nurses older than 31 years coped better with death. Professionals with an optimal self-perception showed significantly lower scores on all personality dimensions evaluated, while a higher level of the anxiety trait predicted worse coping. Although with medium explanatory power, psychoticism, anxiety, and fear of death were the main predictors of the development of optimal coping with death among Spanish nurses. These characteristics together with information from the work environment and evidence-based practice could help to develop better routines and contexts of care for nurses working in end-of-life care.

## 1. Introduction

Caring for terminal patients and their bereaved families and self-competence with death are considered major emotional challenges in the practice of nursing professionals [1]. Health professionals are focused much more on saving lives or curing an illness than caring for the needs and wishes of the dying patient; thus, providing high-quality care in end-of-life (EoL) care requires having the correct attitude, education, and training in the basic principles of palliative care [2]. Furthermore, it has been confirmed that unrealistic expectations for providing optimal care to dying patients may be associated with guilt and dissatisfaction among professionals, endangering their well-being and the quality of care [3,4]. Individual factors, such as age, gender, cultural background, religious beliefs, professional experience, and coping skills, may have an impact on the perception of death, attitudes, and behaviors towards death [5], as well as death anxiety [6]. According to the terror management theory, people create the need for a positive worldview or a sense of their own meaning in life in order to accept their own inevitable demise and guard themselves against death anxiety [7]. Thanks to advances in medicine, healthcare professionals may forget that a patient’s death is not the result of a poor clinical outcome; therefore, if nurses do not accept death as a part of life, this may influence their emotions and behaviors in dealing with dying patients [8]. Fear also modifies attitudes towards the care of dying patients [5]. Ultimately, EoL care involves personal growth and includes several aspects of personality that have been linked to coping with complex stressful emotional situations, such as death [9], and which act as buffering mechanisms for the stress generated [10,11].

### 1.1. Background

In situations involving the end of life, coping is a concept related to stress and anxiety control. Differentiating between the perception of coping and control over the environment is important, since coping tries to manage stress-generating situations to cope with, minimize or tolerate everything that exceeds the limits of a person through their actions, thoughts and emotions. This coping is part of a person’s personal psychological or psychosocial resources, so personality traits exert a mediating effect on coping [10].

The death of a patient or its proximity in the workplace, without the ability to make patients well, may put the emotional and psychological well-being of the professional at risk [7] and negatively compromise the quality of care or professional care and effectiveness of caring for other patients [12], particularly in highly specialized settings [13].

Previous studies have suggested greater emotional exhaustion, distancing, and depersonalization in nurses with greater fear and avoidance attitudes towards death [14], while greater clinical experience and a high degree of caregiving knowledge are important factors contributing to less emotional impact in end-of-life situations [15]. Regardless of the setting, nurses who rely on their coping strategies and skills in stressful work situations may cope better with death and be more effective in performing their work [16]. In addition, both the psychological health and the quality of life of nurses also appear to have a significant effect on the provision of quality palliative care [17], which justifies the interest in understanding how these factors affect nurses’ perceptions when faced with dying patients. This information may be essential for nursing managers, as it would enable them to strengthen coping efforts by minimizing the emotional impact and promoting nurses’ well-being, leading to quality nursing care at the end of life [18].

The present study aimed to assess the self-perceived competence in coping with patients’ death among Spanish nurses from different care settings and to investigate its relationship with their personality traits, anxiety, and fear of dying and death through validated questionnaires.

### 1.2. Hypothesis

According to several previous studies, the psychological health of a nurse may impact specific their self-perceived competence in end-of-life processes as well as the quality of palliative care provided [3,5,7,13]. However, little is known about how the personality or personality traits of Spanish registered nurses relate to self-perceived competence in coping with a patient’s death, although it is expected that these aspects also influence this specific competence.

## 2. Methods

### 2.1. Design and Setting

A cross-sectional study was designed to include registered nurses (RNs) from all the professional nursing associations in Spain (one association for each of the 50 Spanish provinces), working in different healthcare institutions (ambulatory wards or hospitals and general or specialized care settings (ICU, palliative care, emergencies, surgery, psychiatric units, etc.)) and with different complexities of care.

### 2.2. Participants

Convenience sampling of Spanish nurses was performed via a web survey between February 2015 and April 2016. The sample size was calculated based on the total population of Spanish registered nurses (274,817 professionals) on 31 December 2014 (National Institute of Statistics, Madrid, Spain) and to obtain a statistical significance of *p* < 0.05 (requiring at least 384 participants). Complete information was collected from a total of 534 Spanish nurses.

### 2.3. Data Collection

All the Spanish nursing associations sent an invitation letter to their members together with the study information and a consent form that included both the objective of the study and the use and treatment of the data obtained. All professional nurses were invited to participate in a freely accessible survey through the website of each association. Consenting nurses also received, by email, a unique web link to the online survey. Participants completed several questionnaires to obtain information regarding their socio-demographic background and attitudes to death in a Google Drive database. The inclusion criteria were to be older than 18 years; have working experience in health institutions, regardless of the clinical care environment; belong to a Spanish professional nursing college and be able to read and write in Spanish and have access to the Internet. No exclusion criteria were established.

Socio-demographic and occupational information (age, sex, level of education (undergraduate and postgraduate), years of nursing experience, work setting (primary or home care; general hospital care; specialized hospital care; and critical care or emergency care) and type of health institution (public, private, or co-public)) was collected through an ad hoc questionnaire, while outcome and predictor data were gathered using specific validated questionnaires among the Spanish population.

### 2.4. Instruments

The outcome was measured with the Coping with Death Scale (CDS) [19], devised to measure the level of self-competence in handling patients’ death and knowledge concerning preparedness for death. The Spanish version from Schmidt-RioValle and colleagues (2012) [16] was used. This questionnaire consists of 30 items related to understanding, expression of emotions and communication with or help for dying patients (e.g., “I feel able to handle the death of others close to me”, “I can express my fears about dying”, and “I can help people with their thoughts and feelings about death and dying”), quantified by a Likert scale with seven response options (from 1 (strongly disagree) to 7 (totally agree)), with a score range from 30 to 210. A total score value below 105 indicates inadequate coping, while a score greater than 157 represents optimal coping. This questionnaire has shown good internal consistency with a Cronbach’s alpha value of 0.893 among nurses working in oncology services [20].

As predictors of the nurses’ self-perceived competence in coping with death, some personal and emotional characteristics were selected and measured using the following validated questionnaires:

(1) Eysenck Personality Questionnaire Revised-Abbreviated (EPQR-A). The EPQR-A is an abbreviated form of the Eysenck Personality Questionnaire [21], consisting of 24 items that assess three personality subscales (extraversion (E), neuroticism (N) and psychoticism (P)) and a lie subscale. Each scale consists of 6 questions, scored yes (1) or no (0). The first three subscales measure personality traits, while the latter evaluates the level of sincerity. A high E score corresponds to an outgoing, talkative, energetic, assertive, and gregarious person, indicative of a greater presence of extraversion. A high N score implies an anxious, worrying individual who is moody and frequently depressed and finds it difficult to return to a calmer state after each emotionally arousing experience. A high P score defines a personality type that is prone to take risks and might engage in antisocial or non-conformist behavior. The lie subscale assesses the tendency to issue responses of social desirability; a score greater than 4 would void this test. The Spanish version of this questionnaire, used in this study, showed adequate temporal stability and alpha coefficients for the extraversion, neuroticism and psychoticism subscales (0.84, 0.75, and 0.50, respectively) [22].

(2) State–Trait Anxiety Inventory (STAI). The STAI assesses the presence and severity of current symptoms of anxiety and a generalized propensity to be anxious. The STAI scale includes two components: a personality factor (anxiety trait), defined as the general tendency of the individual to perceive situations as more threatening, and a second factor (anxiety state) that evaluates relatively stable aspects of “anxiety proneness”, including general states of calmness, confidence, and security [23]. In this study, only the anxiety trait component was evaluated, consisting of 20 items with a 4-point Likert response scale, assessing the frequency of feelings “in general”: (1) almost never, (2) sometimes, (3) often, and (4) almost always. The total score was obtained by adding the values of the items (after reversing the scores on the negative items), with higher scores corresponding to greater levels of anxiety detected. The Spanish version used here [24] has also shown good internal consistency (α values between 0.94 and 0.98) and stability (r-values between 0.81 and 0.93), both in general and clinical populations [25].

(3) Collett–Lester Fear of Death Scale (CL-FODS) [26]. The CL-FODS is a classic multidimensional instrument that distinguishes between death and the process of dying for oneself and others and is used in assessing attitudes toward death [27,28]. The CL-FODS includes 28 items structured in 4 subscales with 7 items each: (1) fear of death of self (such as dying young); (2) fear of dying of self (such as the degeneration entailed in the process of dying); (3) fear of death of others (such as the loss of a loved one); (4) fear of dying of others (such as accompanying someone who is dying). All subscales are quantified by a 5-point Likert scale (from 1 (nothing) to 5 (much)), with higher scores meaning greater fear of death or dying. By calculating the average of the respective responses, the total score and the scores for each subscale are obtained. Total scores range from 28 to 140, and the score in each subscale ranges from 7 to 35; higher scores denote greater fear of death or death anxiety. The Spanish version from Tomás-Sábado et al. (2007) was used, which has also been shown to have adequate internal consistency [27].

### 2.5. Data Analysis

A descriptive statistical analysis of the socio-demographic data of the sample was performed. The relationship between quantitative variables was calculated using the Pearson correlation coefficient, and the Kruskal–Wallis and Mann–Whitney U-tests were used to compare variables that did not follow a normal distribution. Furthermore, a simple linear regression analysis was performed to calculate the individual predictive value of each of the dimensions evaluated in the Coping with Death Scale. Finally, multiple linear and logistic regression models were applied using the stepwise procedure to select the best predictors of the perception of coping with death. All analyses were performed with a statistical significance of *p* < 0.05, showing the confidence interval (CI) associated with each parameter. The statistical package SPSS version 23 was used for data analysis (SPSS Inc., Chicago, IL, USA).

### 2.6. Ethical Considerations

The study was approved by the Regional Committee of Bioethics and authorized by the Professional College of Nursing of Granada, as well as by all the Ethics Committees of the participating professional nursing colleges. The study followed the principles of the Helsinki Declaration of 1975. All nurses participated individually and anonymously in accordance with the national legislation on Personal Data Protection (Law 3/2018) and provided all required information through the validated questionnaires.

## 3. Results

Most of the participating nurses were women (78.7%), with a mean age of 39.7 years (range 22 to 65 years), a graduate degree (66%) and working in non-private healthcare institutions (77%)—mainly in primary care centers (27.7%) or general hospitals (27.7%). One-third (38%) of the subjects had extensive work experience (>20 years) as nurses. More than half of the nurses (61.2%) reported an optimal self-perceived ability to cope with death (>157 points). The socio-demographic characteristics of the study population in relation to self-perception of coping with death are shown in Table 1. The findings indicate that gender, older age, work experience, and educational background are statistically significantly associated with death competence scores. In particular, males showed higher scores than women did (*p* = 0.006), and younger nurses or those with less than 10 years of nursing experience reported lower self-perceived competence compared to older nurses (*p* < 0.001) (Table 1).

Among our study population, gender and professional experience were significantly correlated variables (*p* = 0.04). In total, 64% of the female participants had less than 10 years of professional experience, while 70% of the males reported more than 20 years of experience (data not shown).

The resulting Fear of Death Scale total scores among the professional nurses were moderately high. Most of the four CL-FODS subscales scored very similarly: “fear of death of others” (23.6 ± 4.0), “fear of the process of dying of self” (21.5 ± 4.1), and “fear of the process of dying of others” (22.8 ± 7.6), except the subscale “fear of death of self” (19.6 ± 3.8), which scored lower than the other subscales. Regarding personality, the average extraversion subscale score obtained was double (4.2 ± 0.9) that obtained in the other three subscales of the EPQR-A personality questionnaire (neuroticism, 2.1 ± 0.9; psychoticism, 2.8 ± 1.3; lie, 2.4 ± 0.9). Participants also reported low levels of anxiety (29.0 ± 5.2) (Table 2).

Pearson’s correlation coefficient showed that age (r = 0.182, *p* < 0.001) and the four subscales of the CL-FODS were significantly and positively correlated with optimal self-perception of coping with a patient’s death (fear of death of self: r = 0.356; fear of death of others: r = 0.405; fear of the process of dying of self: r = 0.293; fear of the process of dying of others: r = 0.556; all *p* < 0.001). However, significant negative relationships were found for two of the personality traits, namely neuroticism (r = −0.167, *p* < 0.001) and psychoticism (r = −0.221, *p* < 0.001), as well as for anxiety trait (r = −0.239, *p* < 0.001). The multivariate contrast (Tukey’s pairwise comparison) also showed a significant effect of the dimensions of the EPQR-A questionnaire on the perception of coping with death, with nurses showing an optimal coping perception having lower scores in the neuroticism and psychoticism dimensions compared to those with an inadequate self-perception of coping. In the same way, participants who perceived their coping with death as inappropriate had higher significant levels of anxiety than shown by those with optimal coping. Finally, participant nurses with an optimal self-perception of coping with death also showed lower scores in total and for all four dimensions of the CL-FODS, with the total score and the dimension of fear of death of others, reaching statistical significance (Table 3).

The multivariate linear regression model revealed that some of the selected predictors, including fear of death (total score), psychoticism, and anxiety trait, were related to the nurse’s perceived end-of-life coping. Thus, negative associations were found for the CL-FODS total score, the psychoticism subscale and anxiety trait, while age was positively associated with self-perceived coping with death. This linear regression model explained 10.3% of the variance and showed that the factor with the highest predictive capacity was the level of psychoticism (β = −3.19, *p* = 0.003), with a lower level of psychoticism behavior indicating a greater level of self-perceived coping with the dying patient (Table 4). The multivariate regression logistic model corroborated that higher fear of death, a psychotic personality and a higher level of anxiety decrease ability to cope with death, and that with increasing age, the probability of coping optimally increases by 3% (data not shown).

More complete multivariate linear regression models were developed to predict the self-perceived competence of Spanish nurses regarding patient death and to allow a relative comparison of the magnitude of the association of the different regression coefficients, including all the participants’ information gathered (personality traits, anxiety, fear of death and also information on work experience and environment, evidence-based practice and occupational stress previously analyzed) [28]. The multivariate linear model revealed that more extensive nursing experience and training (more than 10 years) increased the probability of appropriate coping with dying patients, more so than age, work environment (total score), and evidence-based practice characteristics (practice and attitude). However, greater fear of death and higher levels of anxiety and psychoticism remained inversely associated with coping with death. Negative associations were also found for female gender (Supplementary Table S1). The multivariate regression linear model with these variables explained at least 25% of the variance (Supplementary Table S1).

Similarly, when the outcome was dichotomized, the multivariate regression logistic model also revealed that some socio-demographic and work environment characteristics and evidence-based practice, in addition to fear of death and anxiety level, were related to coping with death (data not shown).

## 4. Discussion

This study found that optimal self-perception of professionally coping with death was related to some characteristics or personality traits of nurses, specifically low levels of anxiety, psychoticism, and fear of death, demonstrating interconnections between the emotional factors and death. The findings also indicated that age (>31 years)—and experience—was a significant predictor of death competence score. However, the predictors showed limited explanatory power (10.3%).

Self-perceived competence with death among Spanish nurses was mainly related to a low total level of fear of death, possibly reflecting the role of self and personal preparation in performing death-related work. A positive attitude towards death—that is, acceptance of death—was one factor with a high predictive capacity for coping with death among Spanish nurses. The psychological meaning management theory (MMT) of death acceptance originates from existential-humanistic theory and constructivist perspectives and integrates cognitive-behavioral processes on how to handle various processes to satisfy basic needs for survival and happiness [29]. Thus, a positive attitude towards death indicates less fear and a greater level of self-perceived coping with the dying patient.

We also found that coping with death was negatively and significantly correlated with some personality traits, such as psychoticism, and with anxiety. Pérez-Mengual and colleagues (2021) [30] also observed the negative influence of some personality traits, such as neuroticism and being introverted with little support and social skills, with the tendency to depression, low self-esteem, anxiety, and irritability, or with a low level of self-care and self-awareness. Yu and colleagues (2016) [4], as well as Zheng and colleagues (2021) [31], suggested that excessive and maintained anxiety could negatively impact the coping strategies of EoL careers; in contrast, positive coping resources and high self-esteem, optimism, and self-efficacy were associated with positive work appraisals related to work performance [32]. When anxiety was related to the dimensions of fear of death, greater trait anxiety was associated with less fear of one’s own or another’s death, as well as of one’s own and another’s death process (data not shown). However, other personality traits, such as neuroticism and psychoticism, showed a variable influence depending on the dimension of fear of death studied. Previous studies have also examined the relationship between death anxiety and individual personality characteristics. The revision by Zuccala and colleagues (2019) [33] showed a positive and significant association of neuroticism, as a subscale of emotional stability or instability, with fear and anxiety about death in several of the studies analyzed. Similarly, Pérez-Mengual and colleagues (2021) [30] showed that neuroticism and gender were significantly related to death anxiety, finding that neuroticism is linked with low frustration tolerance [34].

It is widely believed that fear operates by hindering coping, but no studies have been found that analyze the explicit relationship between fear of death and coping with death, probably because both characteristics have been considered synonymous. We were unable to find any studies that had analyzed coping with death in relation to individual personality traits. We believe that this could be due to the lack of precision of these two concepts, which are often used as equivalent terms (fear, anxiety, and coping) in the context of death. Therefore, for future research, it would be necessary to go deeper into the aspects that most influence coping, whether personal or contextual, among nursing professionals dedicated to the care of others in order to have tools to ensure adequate coping among nursing professionals.

Coping competences are acquired through work and personal experience, which increase with age [7,35]. Accordingly, nurses with less than 10 years of experience were more vulnerable and exhausted, had higher levels of depersonalization and had lower levels of successful coping compared to nurses with more years of experience who were able to maintain distance and set boundaries in end-of-life care [1]. Dijxhoorn and colleagues (2021) [36] also indicated that personal maturity and greater professional experience were factors that improved both global and specific competence in coping with death; similarly, Furingsten and colleagues (2015) [37] pointed out that the key to the most appropriate coping strategies was awareness of one’s own needs, strengths, and limitations, finding that professionals with more than 20 years of work experience had the highest coping scores. Nevertheless, other authors found that greater professional experience (ICU workers) was not a guarantee of better coping with death [1]. Some studies have also highlighted the potential value of more comprehensive training in end-of-life care [5], as well as exposure to distressing situations, such as bereavement. Cheung and colleagues (2018) [1] suggested that those who do not have firsthand experience of bereavement and do not have the knowledge and skills to recover and adapt in the face of significant adversity at a level of competence similar to those with greater professional experience [5] may have lower self-competence.

Several previous studies have corroborated that in care for dying patients, men perceive themselves to be more rational, decisive, and resilient [35] and show a pattern of higher level of self-efficacy, self-reliance, and competitiveness in coping with the difficult emotions of dying [17] and maintaining their well-being in nursing practice and experience [34,36]. Women, however, tend to perceive more anxiety in the face of death than men do [38,39]. These gender differences could be due to the greater likelihood of women to develop emotional distress, their greater concern for the well-being of others [40] and also because of the hegemonic patriarchal culture that differentiates different types of emotional and work coping between men and women [30,34]. Women, moreover, would show a greater concern for death as they are the ones who most often care for the dying [41]. In this regard, several emotional effects have been described specifically for nurses, such as compassion fatigue or empathy burnout, related to prolonged exposure to suffering and excessive compassionate stress; exhaustion or burnout due to prolonged exposure to work stress and over-empathy due to affective empathic care and excessive pathogenic guilt [42]. It is, however, necessary to point out that gender was statistically significantly correlated with professional experience among our study population.

Other studied variables also showed some influence in coping with death in the work setting, including the type of healthcare institution. In this regard, the worst level of competence was found among nurses working in private centers, which could be because nurses in these centers were younger, had less work experience, were probably faced with worse working conditions, were generally more insecure and had reduced salaries and shorter contract periods [3,15]. In our study population, we found a strong correlation between the variables of nursing work experience, gender and age, which could explain the lack of association with the response variable (Bugen scale score).

Self-competence in death-related work among Spanish nurses has previously been related with the work setting [28]. Nurse participants who worked in primary care were found to present higher scores for coping (CL-FODS) than those working in “critical care and/or emergency” services or specialized hospital units. Ding and colleagues (2019) [43] pointed out that this could be due to the greater commitment because of the closer professional–patient relationships in primary care, while there are ongoing gaps in specialist palliative care availability within general practices for dying patients.

### Strengths and Limitations

This study has several shortcomings and strengths. The main limitation of the study is its cross-sectional design, which prevents us from establishing causal relationships between the variables analyzed, as well as determining the causal direction of the predictors of death coping competence. Without longitudinal data, it is not possible to demonstrate the temporal precedence of the selected predictors. Secondly, the system of recruitment and data collection through an online survey limits the generalizability and made it difficult to have real control over the participants. The study sample consisted of individuals who chose to respond to the online questionnaires made available from their professional association. This pre-existing interest in the topic possibly biased the death competence scores towards more adaptive levels than would be observed in a representative sample of the Spanish nursing population. It is also possible that the participants may have overestimated their achievements with desirable responses, attributable to their perceived role as “competent professionals” in relation to EoL work. Nevertheless, the selected validated questionnaires have been used in numerous studies among the Spanish population, thus reducing some of the possible biases. Finally, the predictive models analyzed had limited explanatory power, indicating that coping with death among Spanish nurses also depends on other factors that should be studied. The large sample size and the participation of nurses from different regions of Spain and with varying levels of competence and experience in the care of the dying are the main strengths of this study. Moreover, most of the participants were women, which coincides with the gender distribution in the nursing profession in Spain.

## 5. Conclusions

The results of this exploratory study may provide a basis for understanding the relationship between coping competence and the personal and emotional characteristics of nursing professionals, although they should be interpreted with caution. Knowledge of the influence of these characteristics on the capability to cope with death will help to develop better care routines and reduce the underlying emotional burnout for nurses working in EoL care, improve their quality of care and ultimately change their perception of adequate self-competence, which, in turn, will improve the quality of patient care. Our data suggest that there is room for improvement in the self-competence with death-related work among nursing professionals. Knowledge of the gaps in nursing professionals will also enable the implementation of prevention, behavioral and emotional support programs that will lead to a higher level of self-competence in dealing with a patient’s death.

## Figures and Tables

**Table 1:** Sociodemographic characteristics of the Spanish nurse participants (*n* = 534).

Variables	*n* (%)	Coping with Death Scale		*p*
		Mean	SD	
Age (years)				
≤30	150 (28.1)	128.7	27.6	<0.001
31–42	162 (30.3)	135.8	28.0	
43–54	151 (28.3)	142.9	29.1	
55–65	71 (13.3)	142.1	2.9	
Gender				
Male	114 (21.3)	142.6	28.4	0.006
Female	420 (78.7)	135.0	28.4	
Nursing experience				
<10 years	214 (40.1)	129.9	27.0	<0.001
10–20 years	118 (22.1)	138.2	29.3	
>20 years	202 (37.8)	142.9	28.2	
Academic degree				
Graduate	354 (66.3)	135.1	28.4	0.038
Postgraduate	180 (33.7)	139.7	28.8	
Healthcare setting				
Critical care and emergency	121 (22.7)	135.2	30.8	0.883
General hospitalization unit	117 (21.9)	138.9	27.4	
Specialized hospitalization unit	148 (27.7)	137.5	28.6	
Primary care	148 (27.7)	135.2	27.5	
Sector				
Public	411 (77.0)	138.0	28.8	0.008
Private	90 (16.9)	128.9	27.2	
Co-public	33 (6.2)	141.4	26.3	

**Table 2:** Subscale scores on the CL-FODS, EPQR-A, and STAI-T questionnaires among Spanish nurses (*n* = 534).

Variables	Mean	SD	IQR	
CL-FODS (Total score)	80.6	9.6	74.0	87.0
Fear of death of self	19.6	3.8	17.0	22.0
Fear of dying of self	21.5	4.1	19.0	24.0
Fear of death of others	23.6	4.0	21.0	26.0
Fear of dying of others	22.8	7.6	16.0	30.0
EPQR-A				
Extraversion	4.2	0.9	4.1	4.3
Neuroticism	2.1	1.3	2.0	2.2
Psychoticism	2.8	1.2	2.7	2.9
Lie	2.4	0.9	2.3	2.5
STAI-T				
Anxiety trait	29.0	5.2	28.6	29.4

Abbreviations: CL-FODS: Collett–Lester Fear of Death Scale; EPQR-A: Eysenck Personality Questionnaire Revised—Abbreviated; STAI-T: State–Trait Anxiety Inventory; IQR: interquartile range.

**Table 3:** Association between coping with death and the EPQR-A, CL-FODS, and STAI results among Spanish nurses (*n* = 534).

Variables	Inadequate Coping (*n* = 107)		Optimal Coping (*n* = 327)		
	Mean	SD	Mean	SD	*p*
Age (years)	36.3	10.5	40.9	11.3	**0.002**
CL-FODS (total score)	82.3	8.8	79.3	8.9	**<0.001**
Fear of death of self	19.5	3.4	18.8	4.0	0.818
Fear of dying of self	21.2	4.1	21.0	3.9	0.087
Fear of death of others	24.1	4.1	22.5	4.0	**0.030**
Fear of dying of others	23.3	7.9	21.9	7.6	0.165
EPQR-A					
Extraversion	4.3	0.8	4.2	1.0	0.161
Neuroticism	2.4	1.4	2.0	1.3	**<0.01**
Psychoticism	3.1	1.3	2.6	1.1	**<0.001**
Lie	2.5	0.9	2.4	0.9	0.120
STAI-T					
Anxiety-trait	30.2	5.0	28.3	5.1	**<0.001**

Abbreviations: CL-FODS: Collett–Lester Fear of Death Scale; EPQR-A: Eysenck Personality Questionnaire Revised—Abbreviated; STAI-T: State–Trait Anxiety Inventory. A total score value lower than 105 indicates inadequate coping, while one greater than 157 indicates optimal coping.

**Table 4:** Multivariate linear regression model of Coping with Death Scale among Spanish nurses (*n* = 534).

Variables	Β	CI	*p*
(Intercept)	185.74	158.58–212.89	**<0.001**
Age (years)	0.28	0.06–0.49	**0.011**
CL-FODS (total score)	−0.26	−0.50–−0.01	**0.043**
Psychoticism	−3.19	−5.30–−1.09	**0.003**
STAI-T (anxiety trait)	−1.05	−1.52–−0.59	**<0.001**

Abbreviations: CL-FODS: Collett–Lester Fear of Death Scale; STAI-T: State–Trait Anxiety Inventory (R^2^: 0.103).

## Data Availability

The data that support the findings of this study are available on request from the corresponding author. The data are not publicly available due to privacy or ethical restrictions.

## Supplementary Materials

The following are available online at https://www.mdpi.com/article/10.3390/ijerph18189515/s1, Table S1: Multivariate regression linear model of Coping with Death Scale among Spanish nurses (*n* = 534).
