# Supplementary material for: Personal and Emotional Factors of Nursing Professionals Related to Coping with End-of-Life Care: A Cross-Sectional Study
Source: Int J Environ Res Public Health. 2021 Sep 9;18(18):9515. doi: 10.3390/ijerph18189515 (PMC8465186; doi:10.3390/ijerph18189515)
Supplement: Supplementary file 1 [file ijerph-18-09515-s001.zip › ijerph-1339781-supplementary.pdf]

Supplementary Table S1. Multivariate regression model of the Coping with Death Scale with all information gathered among Spanish nurses (n=534)

	Model 1			Model 2			Model 3			Model 4		
Predictors	E	CI	p	E	CI	p	E	CI	p	E	CI	P
(Intercept)	120.61	91.87 – 149.35	**<0.001**	110.74	82.80 – 138.69	**<0.001**	123.88	95.98 – 151.77	**<0.001**	115.29	87.02 – 143.55	**<0.001**
Gender (female)	-5.09	-10.39 – 0.22	0.060				-6.13	-11.45 – -0.81	**0.024**	-5.13	-10.45 – 0.19	0.059
Nursing Exp (10-20 years)	4.94	-0.95 – 10.83	0.100	6.21	0.37 – 12.05	**0.037**				5.72	-0.13 – 11.57	0.055
Nursing Exp (> 20 years)	6.65	1.39 – 11.92	**0.013**	8.51	3.43 – 13.59	**0.001**				7.97	2.87 – 13.06	**0.002**
PES-NWI overall score	7.28	2.90 – 11.66	**0.001**	7.34	2.94 – 11.73	**0.001**	7.77	3.37 – 12.17	**0.001**	7.49	3.11 – 11.88	**0.001**
Practice of EBP	0.58	0.25 – 0.92	**0.001**	0.63	0.29 – 0.97	**<0.001**	0.55	0.21 – 0.88	**0.001**	0.60	0.27 – 0.94	**<0.001**
Attitude towards EBP	2.35	1.72 – 2.97	**<0.001**	2.38	1.75 – 3.01	**<0.001**	2.47	1.84 – 3.10	**<0.001**	2.38	1.76 – 3.01	**<0.001**
CL-FODS (total score)	-0.31	-0.54 – -0.08	**0.010**	-0.29	-0.53 – -0.06	**0.014**	-0.32	-0.56 – -0.09	**0.007**	-0.30	-0.53 – -0.06	**0.013**
Psychoticism	-1.94	-3.96 – 0.09	0.061									
STAI-T	-0.84	-1.30 – -0.39	**<0.001**	-0.97	-1.41 – -0.53	**<0.001**	-1.03	-1.47 – -0.59	**<0.001**	-0.96	-1.40 – -0.52	**<0.001**
R^2		0.269			0.259			0.249			0.264	

PES-NWI = Practice Environment Scale of the Nursing Work Index; EBP = Evidence-based Practice; STAI-T: Trait Anxiety Inventory; CL-FODS: Collett-Lester Fear of Death Scale.

Supplementary Table S1 shows several multivariate linear regression models for the Coping with Death Scale among Spanish nurses. Model 1 (full model) included some of the socio-demographic variables (gender, nursing experience) together with information collected about their work environment (PES-NWI global score), their evidence-based practice (Practice and Attitude towards EBP), as well as the personal-emotional characteristics (CL-FODS (total score), Psychoticism and STAI-T) of the participants. Model 2 did not include the variables Gender and Psychoticism, Model 3 did not include the variables Nursing Experience and Psychoticism, and Model 4 only discarded Psychoticism. The explanatory power of the models (R2) varied very little.
